# The Annual Meeting of the Alumni Association of the Baltimore College of Dental Surgery

**Published:** 1877-04

**Authors:** 


### ARTICLE II.

### *The Annual Meeting of the Alumni Association of the Baltimore College of Dental Surgery.*

[Reported for this Journal.]

The Annual Meeting of the Alumni Association of Baltimore College of Dental Surgery, was held in the lecture room of the College, March 9th, 1877. The President, Dr. S. J. Cockerille, of Washington, D. C., on calling the meeting to order, delivered the annual address.

A letter from Dr. W. W. H. Thackston, of Va., of the class of 1842, was read by the Secretary, stating that unavoidable detention—illness in his family— prevented his being present, and that he had forwarded the manuscript of the address he was at the last meeting selected to deliver, to be read. Prof. Gorgas then proceeded to read the paper, [published elsewhere] and at its conclusion, a vote of thanks was unanimously tendered Dr. Thackston for the very able address just heard, and a resolution for its publication adopted.

The Corresponding Secretary, Dr. Wm. B. Wise, of Va., stated that he had sent out a very large number of invitations to this meeting, and was in receipt of letters from many Alumni, stating their hearty endorsement of the association and its objects, and expressing sincere regret at not being able to be present. One of the letters was from Dr. Carlos Gardner, of Madrid, Spain, wishing the society God speed ; another from Dr. Carl F. Wagner, Stuttgart, Germany, expressing like hopes and regrets of absence.

The following resolution was offered by Dr. Cockerille :

Whereas, the good of our profession is our highest aim, be it therefore

*Resolved,* That no member of this Association shall attend any Society or Dental Convention of any kind, in which any person or persons are allowed to occupy seats as members, who have not received diplomas from some reputable Dental College.

This resolution was adopted without debate, and afterwards, on motion of Dr. J. B. Wood, of Va., reconsidered. Considerable debate arose as to the propriety and expediency of such a measure at this stage of dental progress. Dr. Wood thought that such a resolution would shut out from associating with us as dentists, some of the confessedly best men of the profession; men whose interests were as great in the advancement of the cause of dental education as most of those who held diplomas.

Dr. Jno. Allen, of N. Y., the inventor of the "continuous gum" made some objections to the resolution, as too sweeping in its scope. There were many men, he said, in the profession, men at whose feet we might be proud to sit and learn, who had given to dentistry much of its dignity, who had not diplomas, some of them for the simple reason, that at the date of their entering the profession there were no colleges.

Prof. T. S. Latimer, took the ground, that circumstanced as the profession is, it could not afford to take such a step as this, as it amounted simply to cutting ourselves off from participation in organizations, in which, by remaining we might later in their history exercise some controlling influence.

Prof. H. R. Noel took substantially the same ground, urging that it would be unwise to isolate ourselves so early in life as a profession, but that we had better by earnest effort in these societies, and in every way work up the turn of the profession to a higher pitch, and so gradually point the way for such a state of affairs.

The debate was participated in by others, the general impression among those speaking being, that sentiment in the profession was hardly ripe for such an advanced step, and that the societies could hardly afford to dispense with many of those who would be shut out by this resolution.

Prof. Hodgkin offered as an amendment: Provided that such person or persons commenced the practice of dentistry less than twenty (20) years ago; and urged that this amendment would probably relieve the resolution of its objectionable features. It was not intended to strike at the honor of those who had years ago assisted in laying the foundation of our profession, who had grown gray in its service, and wore honor such as any graduate might covet, but it was hoped that some plan could be adopted which would shut out from competition with us, the vast crowd of raw office students, who after a few weeks or months study in the laboratory of some cheap dealer in vulcanite, "set up shop" for themselves, and become in so far as the public discrimination could go by seeing the outside, dentists; the peers apparently, of not only those who had toiled for diplomas, but of those who had won distinction without.

The resolution was finally laid on the table.

Prof. É. Lloyd Howard, who had been announced to deliver an address on "anæsthetics," owing to the lateness of the hour at which he appeared (having been detained by official duties,) stated that he would offer at the banquet at night, some remarks on that subject, and would not now take up the time of the society.

Prof. Hodgkin offered the following resolutions, which were adopted:

*Resolved*, That a committee of three be appointed, whose duty it shall be to draft an "Order of Business" for the expedition of the affairs of this society, such committee to report at the next Annual Meeting. And this committee is hereby empowered to arrange with members of this society and with others, for the reception and reading of papers on subjects of interest to the profession. [Committee, Drs. Howard, Gorgas and Massamore.]

*Resolved*, That a committee of three be appointed, by the President, whose duty it shall be to consider and mature a plan for the organization of some system of Life Insurance among the members of this society. This committee to report at the next Annual Meeting. [Committee, Drs. Hodgkin, Latimer and Davy.]

The following resolution by Dr. Cockerille was adopted:

The President shall appoint a memorial committee, consisting of three members of this society, whose duty it shall be to enquire into the circumstances of the death of any of its members, and make such memorial of the life and services to the profession of such deceased members as may be fitting and proper, such memorial to be put on the records of the society.

The society then proceeded to the business of election of officers for the ensuing year, resulting in the re-election of those at present holding those positions.

The society then adjourned, to meet informally at the Academy of Music, and at the conclusion of the Commencement exercises, proceeded to the Masonic Temple, where a banquet had been provided, and where members discussed the quality of the viands bountifully spread before them, and indulged in the discussions of subjects grave and gay, until the " we sma' hours."

Among the toasts responded to with happiest effect, was one on Profs. Chapin A. Harris, Horace Hayden, Thos. E. Bond, H. Willis Bayley and Washington R. Handy, the founders of American Dentistry, by Dr. Wm. H. Dwinelle, of N. Y., whose reminiscences of the early days of the College and of the prophetic inspirations of Dr. Chapin A. Harris, were exceedingly interesting.

In response to the toast " The Dental Profession—as the discoverer of Anæsthesia, the benefactor of the world :"

Prof. E. Lloyd Howard made a few condensed and forcible remarks on Anæsthetics, showing most clearly that the world owed to the dental profession a debt of gratitude, for the discovery of Anæsthesia; and claiming in the most positive manner for Wells and Morton, the honor of conferring this priceless boon upon suffering humanity. He considered the discovery of chloroform as a matter of course simple, after the way had been found by the application of ether and nitrous oxide gas. The dental profession justly claim, and as a physician, he cordially awarded to them the honor of being the pioneers in this matter.

Prof. H. R. Noel, whose failing health had prevented during the past year, his taking his usual share of college work, was present, and in response to a toast, took the floor in an entertaining reminiscence of his first essay as a teacher of Physiology, and paid an elegant tribute to the memory of Prof. Piggot. He summed up the essentials of success, as being hard work, steady appreciation, continued effort, and gave it as the result of his observation and experience, that no success worth the name was attained in any other way.

Prof. T. S. Latimer, in response to the toast "Woman," paid an eloquent tribute to that ideality to whom all mankind must give homage, and in an address full of poetry, and worshipped adoration of the tender sex, demolished all reasons for his being a bachelor.

Pleasant and appropriate remarks were made by Prof. Thos. Opie, Dean of the college of Physicans and Surgeons, Profs. T. R. Brown, Lynch and Bevan, of that college, and by Drs. John Allen, of N. Y., S. J. Cockerille, and D. McFarlan, of Washington, D. C., Drs. A. J. Volck, T. A. LaFar, W. H. Hoopes and Geo. A. Mills, of Baltimore, Dr. J. B. Wood, of Richmond, Va., C. H. Reynolds, of Canada, M. W. Williams, of Tenn., and others.

At three o'clock, A. M., after having spent a most delightful evening, the society adjourned to meet in the College building, on the morning of the next Commencement Day.

